# Long Covid: a global health issue – a prospective, cohort study set in four continents

**DOI:** 10.1136/bmjgh-2024-015245

**Published:** 2024-10-21

**Authors:** Ekaterina Pazukhina, Esteban Garcia-Gallo, Luis Felipe Reyes, Anders Benjamin Kildal, Waasila Jassat, Murray Dryden, Jan Cato Holter, Allegra Chatterjee, Kyle Gomez, Arne Søraas, Matteo Puntoni, Nicola Latronico, Fernando A Bozza, Michael Edelstein, Bronner P Gonçalves, Christiana Kartsonaki, Oksana Kruglova, Sérgio Gaião, Yock Ping Chow, Yash Doshi, Sara Isabel Duque Vallejo, Elsa D Ibáñez-Prada, Yuli V Fuentes, Claire Hastie, Margaret E O'Hara, Valeria Balan, Tigist Menkir, Laura Merson, Sadie Kelly, Barbara Wanjiru Citarella, Malcolm G Semple, Janet T Scott, Daniel Munblit, Louise Sigfrid, ISARIC Global Covid-19 follow up working group, Adam Ali, ISARICs Clinical Characterisation Group, Laurent Abel

**Affiliations:** 1 ISARIC Global Support Centre, Pandemic Sciences Institute, Nuffield Department of Medicine, University of Oxford, Oxford, UK; 2 Unisabana Center for Translational Science, School of Medicine, Universidad de La Sabana, Chia, Colombia; 3 Clinica, Universidad de La Sabana, Chia, Colombia; 4 Department of Anesthesiology and Intensive Care, University Hospital of North Norway, Tromso, Troms, Norway; 5 Department of Clinical Medicine, Faculty of Health Sciences, UiT The Arctic University of Norway, Tromso, Troms, Norway; 6 Genesis Analytics Pty Ltd, Johannesburg, Gauteng, South Africa; 7 National Institute for Communicable Diseases, Johannesburg, South Africa; 8 Department of Microbiology, Oslo University Hospital, Oslo, Norway; 9 Institute of Clinical Medicine, University of Oslo, Oslo, Norway; 10 UK Health Security Agency, London, UK; 11 Gibraltar Health Authority, Gibraltar, Gibraltar; 12 The Norwegian Corona Cohort, Oslo, Norway; 13 Clinical & Epidemiological Research Unit, University Hospital of Parma, Parma, Italy; 14 Department of Medical and Surgical Specialties, Radiological Sciences and Public Health, University of Brescia, Brescia, Italy; 15 Department of Emergency, Spedali Civili University Hospital, Brescia, Italy; 16 National Institute of Infectious Disease Evandro Chagas, Oswaldo Cruz Foundation, Rio de Janeiro, Brazil; 17 D'Or Institute for Research and Education, Rio de Janeiro, Brazil; 18 Bar-Ilan University The Azrieli Faculty of Medicine, Safed, Northern District, Israel; 19 Department of Comparative Biomedical Sciences, University of Surrey, Guildford, UK; 20 Nuffield Department of Population Health, University of Oxford, Oxford, UK; 21 Department of Internal Medicine No 2, Lugansk State Medical University, Rivne, Ukraine; 22 São João Hospital Centre, Porto, Portugal; 23 Sunway Medical Centre, Bandar Sunway, Selangor, Malaysia; 24 Terna Specialty Hospital and Research Centre, Mumbai, India; 25 University of Oxford Nuffield Department of Medicine, Oxford, UK; 26 Universidad de La Sabana, Chia, Colombia; 27 Long COVID Support, London, UK; 28 The Center for Communicable Disease Dynamics, Department of Epidemiology, Harvard TH Chan School of Public Health, Harvard University, Cambridge, Massachusetts, USA; 29 Pandemic Sciences Institute, University of Oxford, Oxford, UK; 30 NIHR Health Protection Research Unit in Emerging and Zoonotic Infections, Institute of Infection, Veterinary, and Ecological Sciences, University of Liverpool, Liverpool, UK; 31 Liverpool Institute for Child Health and Wellbeing, Alder Hey Children’s Hospital, Liverpool, UK; 32 MRC, University of Glasgow Centre for Virus Research, University of Glasgow, Glasgow, UK; 33 RD&I and COVID Recovery Service, NHS Highland, Inverness, UK; 34 Division of Care in Long Term Conditions, Florence Nightingale Faculty of Nursing, Midwifery and Palliative Care, King's College London, London, UK; 35 Department of Paediatrics and Paediatric Infectious Diseases, Institute of Child’s Health, I M Sechenov First Moscow State Medical University, Moskva, Moskva, Russian Federation; 36 Policy and Practice Research Group, Pandemic Sciences Institute, University of Oxford, Oxford, UK; 37 Global Collaborative Platform, Oxford, UK

**Keywords:** COVID-19, Global Health, Epidemiology, Public Health, Cohort study

## Abstract

**Introduction:**

A proportion of people develop Long Covid after acute COVID-19, but with most studies concentrated in high-income countries (HICs), the global burden is largely unknown. Our study aims to characterise long-term COVID-19 sequelae in populations globally and compare the prevalence of reported symptoms in HICs and low-income and middle-income countries (LMICs).

**Methods:**

A prospective, observational study in 17 countries in Africa, Asia, Europe and South America, including adults with confirmed COVID-19 assessed at 2 to <6 and 6 to <12 months post-hospital discharge. A standardised case report form developed by International Severe Acute Respiratory and emerging Infection Consortium’s Global COVID-19 Follow-up working group evaluated the frequency of fever, persistent symptoms, breathlessness (MRC dyspnoea scale), fatigue and impact on daily activities.

**Results:**

Of 11 860 participants (median age: 52 (IQR: 41–62) years; 52.1% females), 56.5% were from HICs and 43.5% were from LMICs. The proportion identified with Long Covid was significantly higher in HICs vs LMICs at both assessment time points (69.0% vs 45.3%, p<0.001; 69.7% vs 42.4%, p<0.001). Participants in HICs were more likely to report not feeling fully recovered (54.3% vs 18.0%, p<0.001; 56.8% vs 40.1%, p<0.001), fatigue (42.9% vs 27.9%, p<0.001; 41.6% vs 27.9%, p<0.001), new/persistent fever (19.6% vs 2.1%, p<0.001; 20.3% vs 2.0%, p<0.001) and have a higher prevalence of anxiety/depression and impact on usual activities compared with participants in LMICs at 2 to <6 and 6 to <12 months post-COVID-19 hospital discharge, respectively.

**Conclusion:**

Our data show that Long Covid affects populations globally, manifesting similar symptomatology and impact on functioning in both HIC and LMICs. The prevalence was higher in HICs versus LMICs. Although we identified a lower prevalence, the impact of Long Covid may be greater in LMICs if there is a lack of support systems available in HICs. Further research into the aetiology of Long Covid and the burden in LMICs is critical to implement effective, accessible treatment and support strategies to improve COVID-19 outcomes for all.

**WHAT IS ALREADY KNOWN ON THIS TOPIC**

- Prior to this study, global data on Long Covid were sparse, with most studies focused on high-income countries (HICs).
- Long Covid sequelae identified in previous studies included a wide range of symptoms, with fatigue, breathlessness, cognitive dysfunction and various physical complications.
- The lack of precise case definitions, standardised protocols and tools has hindered a comprehensive understanding of Long Covid and its impact globally.

**WHAT THIS STUDY ADDS**

- This study, employing a collaborative harmonised approach to data collection across four continents, reveals key Long Covid characteristics affecting populations worldwide.
- Although the prevalence was higher in HICs, the individual and broader socioeconomic impact may be higher in resource-limited settings.
- Codevelopment of a standardised protocol using validated tools facilitated implementation in resource-restricted settings during the pandemic.

**HOW THIS STUDY MIGHT AFFECT RESEARCH, PRACTICE OR POLICY**

- Our data highlight the need for further research into underlying aetiologies of Long Covid to inform precise case definitions, trials and evidence-based care pathways to improve equitable COVID-19 outcomes.
- Further, research to identify the impact of Long Covid globally, to inform targeted strategies to support recovery and mitigate wider socioeconomic impact.
- Prepositioned standardised follow-up protocols nested in strategic observational and adaptive trial platforms should be incorporated in epidemics and pandemic preparedness plans.

## Introduction

 The COVID-19 pandemic has caused a substantial burden on healthcare systems and economies worldwide, especially in lower-income and middle-income countries (LMICs),^1 2^ with 774 million confirmed cases globally, 3.42 million in LMICs and 7.02 million deaths worldwide (as of 24 January 2024).^3^ SARS-CoV-2 can cause prolonged sequelae and complications, as previously documented for other infections.^4^,^8^ People affected by long-term sequelae coined the term Long Covid. WHO later defined post-COVID-19 condition/Long Covid as occurring in individuals with a history of probable or confirmed SARS CoV-2 infection, usually 3 months from the onset of COVID-19 with symptoms that last for at least 2 months and cannot be explained by an alternative diagnosis, generally impacting on functionality.^9^ Since then, a number of studies have documented a broad spectrum of physical and psychological postacute COVID-19 sequelae affecting both adults and children.^10^ It is estimated that 6%–10% of adults are affected by Long Covid following SARS-CoV-2 infection.^11^ Post-COVID-19 sequelae affect adults of all ages across acute disease spectra.^12^ Hospitalisation has been identified as a risk factor for prolonged COVID-19 recovery.^12^Due to heterogeneous study designs, case definitions, limited research resources and most studies being concentrated in high-income (HICs) countries, underlying aetiology, precise clinical characteristics, optimal care, the global burden and the wider individual and socioeconomic impact remain uncertain. While studies have shown that COVID-19 vaccination can provide protection against Long Covid, the protection is imperfect.^13 14^ Limited access to treatment trials has led to people resorting to trialling non-evidence-based experimental Long Covid treatments, with associated risks and costs to individuals, creating moral and ethical dilemmas for clinicians and challenges for trial implementation.

Despite the many studies exploring Long Covid characteristics, the lack of precise case definitions and heterogeneity in study design and outcome measures have been a challenge for meta-analyses.^15^ Key commonly reported Long Covid sequelae identified include but are not limited to, fatigue, breathlessness, cognitive dysfunction and cardiovascular, muscular and endocrinological complications.^16 17^ A systematic review including 39 COVID-19 follow-up studies identified 60 physical and psychological signs and symptoms, with 41% of patients reporting weakness, 31% fatigue, 26% concentration difficulties and 25% breathlessness.^15^ The review did not identify any studies set in LMICs. LMICs might not have sufficient research or surveillance infrastructure to accurately report the magnitude and effect of COVID-19. Limited access to surveillance and healthcare systems may hide a high burden of Long Covid.^18^ To address this knowledge gap, we convened an International COVID-19 Global Follow-up Working Group of diverse clinical specialists, researchers and Long Covid patient group representatives to develop a standardised, open-access Global COVID-19 follow-up protocol and case report form (CRF) for implementation in any resourced setting during the pandemic.^19^,^22^ The primary aim was to characterise Long Covid, explore risk factors for developing Long Covid and the impact on daily activities and quality of life (QoL) in different regions. The data are relevant for informing strategies and policies to improve equitable long-term COVID-19 outcomes globally.

## Methods

This is a prospective, observational, international cohort study set in 25 sites in 17 countries (Brazil, Colombia, France, The Gambia, Gibraltar, India, Israel, Italy, Malaysia, Norway, Portugal, Russian Federation, South Africa, Spain, Sudan, Ukraine and the UK). The sites were identified through an open call to sites to collaborate via the International Severe Acute Respiratory and emerging Infection Consortium’s (ISARIC’s) clinical research network, website, newsletter and social media and included sites with a capacity to follow up with participants post-COVID-19 hospital discharge. Adults admitted to the hospital due to or identified with SARS-CoV-2 infection during hospital admission were eligible to participate. Participants were contacted at 2 to <6 months and/or 6 to <12 months postdischarge for a follow-up assessment using ISARIC’s Global COVID-19 follow-up protocol and electronic CRF. We established a diverse, inclusive, open-access ISARIC follow-up working group in May 2020, consisting of international multidisciplinary specialist physicians, researchers, public health professionals and people living with Long Covid and developed the protocol through a series of meetings and email iterations.^22^ The CRF was piloted in three countries before being finalised as described previously. The CRF was designed to facilitate implementation in any resourced setting by focusing on critical key data, using validated tools and enabling a combination of clinician-led assessments (in-clinic or by phone) and patient self-assessment via (post and online) to support wide dissemination and uptake during the pandemic restrictions. The data were entered into a Research Electronic Database Capture system (REDCap, V.8.11.11, Vanderbilt University, Nashville, Tenn^23^) and linked with data documented previously during the acute COVID-19 admission collated by the ISARIC Clinical Characterisation Group (online supplemental file) for the analysis. Data were shared with ISARIC’s Global Support Centre at the University of Oxford for integration into a central dataset. Each site investigator was responsible for ensuring local ethical and regulatory approvals and conducting the study on-site.

### Study population

Patients aged 18 years and over admitted to a participating hospital between February 2020 and April 2022 were included in this analysis. Participants were identified via convenience sampling. Patients with laboratory or clinically confirmed COVID-19 diagnosed on or during admission, who consented to be contacted for a posthospital follow-up, for whom a valid contact was provided and who were discharged at least 60 days ago, were eligible for inclusion. We included patients with laboratory or clinically diagnosed COVID-19 since laboratory confirmation depended on the local availability of PCR testing. Any patients with demographic data and at least one answer to the follow-up questions were included in the analysis.

### Variables and outcomes

Explanatory variables at the time of hospital admission, including age, sex and pre-existing comorbidities, were recorded in the acute CRF,^24^ and the data captured during the follow-up assessment in the linked follow-up CRF.^22^ Patients receiving ICU care, high-flow nasal cannulation, non-invasive respiratory support, invasive ventilation and/or vasopressors, were defined as having severe acute COVID-19 as described previously.^1925^,^27^ The primary outcome was self-reported recovery at 2 to <6 or 6 to <12 months following hospital discharge. Secondary outcomes included new or persistent symptoms, breathlessness, fatigue, impact on daily functioning and quality of life (QoL). Breathlessness was measured using the Medical Research Centre (MRC) dyspnoea scale, which grades the effect of breathlessness on daily activities. This 5-point scale measures perceived respiratory disability, with 0 representing no breathlessness and 4 representing an inability to undertake activities of daily living due to breathlessness. Fatigue was measured on a 1–10 Visual Analogue Scale (VAS) where 0 represents no fatigue and 10 represents the worst possible fatigue. The impact on functionality, psychosocial complications and QoL was measured using the EuroQol 5-Dimension 5-level (EQ-5D-5L) instrument. This tool covers five dimensions: mobility, self-care, usual activities, pain/discomfort and anxiety/depression. The person indicates his/her health state for each of the five dimensions. New and persistent symptoms not present prior to their COVID-19 illness were categorised into organ systems and systemic symptoms: (a) Cardiological: palpitations; (b) Dermatological: skin rash, Covid toes; (c) Fatigue; (d) Fever (measured or self-reported), (e) Gastrointestinal: abdominal pain, diarrhoea, constipation, vomiting/nausea; (f) Musculoskeletal: muscle aches/joint pain, ankle swelling; (g) Neurocognitive: headache, problems with sleeping, altered consciousness/confusion, dizziness, tinnitus, problems with balance, paraesthesia, tremor, problems with speaking or communicating, fainting/blackouts, loss of sensation, seizures; (h) Respiratory: cough, cough productive, cough non-productive, shortness of breath, pain in breathing, chest pain and (i) Sensory: lost or altered sense of smell, lost or altered sense of taste (online supplemental file 1: box S1). Patients with at least one symptom group affected at the first follow-up assessment, which was not present prior to COVID-19 onset, were categorised as Long Covid in line with WHO’s definition.^28^

### Statistical analysis

Categorical data were expressed as counts and percentages, and continuous data as median (25th–75th percentiles). We described the frequencies of the outcomes across demographic groups (age categories and sex), comorbidities and acute disease severity. We grouped all measures by time interval (2 to <6 months and 6 to <12 months postdischarge). Countries were categorised as LMICs or HICs based on the World Bank country income classification^29^ (HICs included France, Gibraltar, Israel, Italy, Norway, Portugal, Spain and the UK; LMICs included Brazil, Colombia, The Gambia, India, Malaysia, Russian Federation, South Africa, Sudan and Ukraine). To test for differences across groups, we used a two-proportion z-test. Confidence for the means was calculated using a bootstrap procedure with 1000 iterations group. Risk factors were evaluated using logistic regression, with country income included as a separate covariate. The sensitivity analysis for logistic regression is included in online supplemental file 6 table S3. Other covariates were age, gender, comorbidities and severity at hospitalisation. For breathlessness, we calculated the change in value reported by participants at the follow-up assessment versus before COVID-19 onset. For the health state at the follow-up assessment, we used the EQ-5D-5L tool with the English standardised valuation study protocol value set on the composite time trade-off valuation (https://euroqol.org/publications/user-guides). Overall changes in summary health index, before and after COVID-19 onset, were summarised for the cohort using the Paretian Classification of Health Change method. Summary of the EQ-5D-5L indices index was measured on a scale of 0–1, with 0 reflecting the worst health imaginable; 1 reflecting perfect health. We calculated overall estimates and estimates for individual EQ-5D-5L dimensions, as well as changes in the summary index and individual dimensions. All data processing and statistical analysis were performed using secure R V.4.2.0 (R Foundation for Statistical Computing, Vienna, AUT), with the tidvverse, readxl, ggplot2, gtsummary, reshape and forcats packages using SPSS V.29 (IBM). Scales were transformed into binary variables. For fatigue intensity (10-point VAS), scores ≤4 were classified as 0 (none-to-mild, and scores ≥5 as 1 (moderate-to-severe). Incomplete recovery was measured on a 5-point Likert scale converted to numerical values. Values 1–2 (agree and strongly agree) were categorised as 1 (not completely recovered), values 4–5 (disagree and strongly disagree) categorised as 0 (recovered) and value 3 (neither disagree/agree) as unknown. For breathlessness (initial scales 0–4), the values ≤2 were classified as 0 (none-to-mild breathlessness); values >2 as 1 (moderate-to-severe). The components of EQ-5D-5L (initial range 1–5) were classified as 0 (none-to-mild problems) if the initial value was ≤2 and for values >2 as 1 (moderate-to-severe problems).

### Public and patient involvement

Patients and people living with Long Covid, including the founding members of the UK Charity, Long Covid Support, were involved in informing the CRF design and in the interpretation and representation of the data and review of the manuscript. The CRF was piloted with patients presenting in clinics in three countries and the feedback incorporated into the final version. This included suggestions on the data on symptoms collected, how questions were asked and the patient information.

## Results

**Figure 1:**
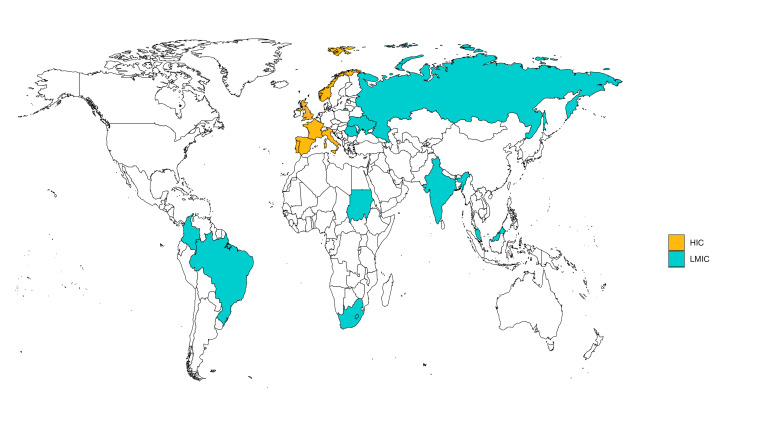
Geographical distribution of participating sites. The map shows the distribution of the participating sites in Brazil, Colombia, France, The Gambia, Gibraltar, India, Israel, Italy, Norway, Malaysia, Portugal, Russian Federation, South Africa, Spain, Sudan, Ukraine, and the UK, and their country income classification. HIC, high-income country; LMIC, low-income and middle-income country.

Of 35 637 participants included in the follow-up studies, 11 860 met the inclusion criteria for this analysis. Of these, 64.3% (7625/11860) were assessed at 2 to <6 months, 55.0% (6520/11860) at 6 to <12 months postdischarge; 19.3% (2285/11860) were assessed at both time points (online supplemental file 2: figure S1: Strengthening the Reporting of Observational Studies in Epidemiology flow chart). 56.5% (6698/11 860) were included in sites in HICs, and 43.5% (5162/11 860) in LMICs sites, in countries in Africa (18.8%, 2228/11 860), Asia (15.8%, 1874/11 860), Europe (60.2%, 7141/11 860) and South America (5.2%, 617/11 860) (figure 1, online supplemental file 3: table S1). The median age was 52.0 (IQR: 41–62) years, 52.1% (6147/11860) were female and 48.5% (5751/11860) were of white ethnicity.

### Pre-existing risk factors

Most participants (87.3%) were classified as having had moderate acute disease. Hypertension (22.4%, 2659/11 860), diabetes mellitus (10.0%, 1187/11 860) and chronic cardiac disease (6.0%, 707/11 860) were the most common comorbidities. 10% (1190/11860) were obese, and 12.7% (1507/11860) were classified as having had a severe illness during acute hospitalisation (table 1). The participants in LMICs and HICs had a similar age distribution, while those included in LMICs had higher rates of pre-existing hypertension, chronic cardiac disease, HIV and obesity. In contrast, HICs reported a higher prevalence of asthma, chronic pulmonary disease and rheumatological disorders.

**Table 1:** Demographic and clinical cohort characteristics stratified by time point and income classification

Characteristic	N=11 860	2 to <6 months assessment		6 to <12 months assessment	
		HIC N=4680	LMIC N=2945	HIC N=2693	LMIC N=3827
Age	52.0 (41.0, 62.0)	52.0 (41.0, 62.2)	52.0 (41.0, 62.0)	52.0 (40.0,63.0)	53.0 (42.0,62.0)
Sex at birth					
Female	6147 (51.8%)	2529 (54.0%)	1447 (49.1%)	1416 (52.6%)	1868 (48.8%)
Male	5655 (47.7%)	2121 (45.3%)	1498 (50.9%)	1248 (46.3%)	1957 (51.1%)
Unknown	58 (0.5%)	30 (0.6%)	0 (0%)	29 (1.1%)	2 (0.1%)
Ethnicity					
Asian	1297 (10.9%)	163 (3.5%)	619 (21%)	120 (4.5%)	568 (14.8%)
Black	1499 (12.6%)	97 (2.1%)	1159 (39.4%)	78 (2.9%)	1042 (27.2%)
Latin American	475 (4.0%)	195 (4.2%)	144 (4.9%)	54 (2%)	108 (2.8%)
Other ethnicities	265 (2.2%)	158 (3.4%)	3 (0.1%)	148 (5.5%)	3 (0.1%)
White	5751 (48.5%)	3325 (71%)	812 (27.6%)	1981 (73.6%)	701 (18.3%)
Unknown	2573 (21.7%)	742 (15.9%)	208 (7.1%)	312 (11.6%)	1405 (36.7%)
Acute COVID-19 severity*					
Moderate	10 353 (87.3%)	4176 (89.2%)	2360 (80.1%)	2255 (83.7%)	3258 (85.1%)
Severe	1507 (12.7%)	504 (10.8%)	585 (19.9%)	438 (16.3%)	569 (14.9%)
Days from hospital admission to follow-up	164.5 (126.0, 239.0)/6815	132.0 (102.0, 162.0)/2632	140.5 (107.0, 165.5)/2,821	249.0 (226.0,284.0)/805	271.0 (241.0, 282.5)/557
Pre-existing comorbidities and risk factors					
Asthma	798/11 860 (6.7%)	433/4680 (9.3%)	97/2945 (3.3%)	255/2693 (9.5%)	162/3827 (4.2%)
Chronic cardiac disease	707/11 860 (6.0%)	237/4680 (5.1%)	125/2945 (4.2%)	151/2693 (5.6%)	314/3827 (8.2%)
Chronic pulmonary disease (not asthma)	463/11 860 (3.9%)	239/4680 (5.1%)	32/2945 (1.1%)	146/2693 (5.4%)	127/3827 (3.3%)
Diabetes	1187/11 860 (10%)	385/4680 (8.2%)	336/2945 (11%)	257/2693 (9.5%)	454/3827 (12%)
HIV	377/11 860 (3.2%)	7/4680 (0.1%)	103/2945 (3.5%)	3/2693 (0.1%)	338/3827 (8.8%)
Hypertension	2650/11 860 (22%)	740/4680 (16%)	866/2945 (29%)	424/2693 (16%)	1344/3827 (35%)
Obesity	1190/11 860 (10%)	234/4680 (5.0%)	534/2945 (18%)	130/2693 (4.8%)	769/3827 (20%)
Rheumatological disorder	429/11 860 (3.6%)	272/4680 (5.8%)	11/2945 (0.4%)	158/2693 (5.9%)	47/3827 (1.2%)
Tuberculosis	34/11 860 (0.3%)	4/4680 (<0.1%)	23/2945 (0.8%)	2/2693 (<0.1%)	20/3827 (0.5%)

The respondents (n=2285) who followed up at both intervals are included under each assessment time point.

* Acute severity is defined as receiving any of the following during the acute hospitalisation: Intensive care unit (ICU), High-flow nasal cannula (HFNC), Non-invasive respiratory support, Invasive ventilation, Vasopressors.

HIC, high-income country; LMIC, low-and middle-income country.

### Medium-term and longer-term impact on physical and psychosocial health

The most commonly reported symptoms were fatigue/malaise, headache, shortness of breath and muscle/joint pain at both follow-up assessments, with a higher frequency reported in HICs versus LMICs (online supplemental 4: figure S2). Of the participants, 37.0% (2400/6402) reported not feeling fully recovered at 2 to <6 months and 46.5% (2863/6152) at 6 to <12 months postdischarge. (online supplemental file 5: table S2). At the 2 to <6 months assessment, participants in HICs were significantly more likely to report moderate-to-severe fatigue (42.9 vs 27.9%, p<0.001), feeling not fully recovered (54.3% vs 18.0%, p<0.001), fever (19.6% vs 2.1%, p<0.001) and have at least one persistent or new symptom (69.0% vs 45.3%, p<0.001) compared with those in LMICs. The proportion reporting anxiety/depression (9.2% vs 5.6%, p<0.001), pain/discomfort (11.6% vs 6.7%, p<0.001) and problems doing usual activities (10.0% vs 3.5%, p<0.001) was also higher in HICs compared with LMICs. In contrast, there were no significant differences in the proportion of moderate-to-severe breathlessness across settings and time points, ranging from 8.4% to 11.8%.

Similar differences were observed at the later follow-up assessment (6 to <12 months), with a higher proportion of participants in HICs reporting fatigue (41.6% vs 27.9%, p<0.001), not feeling fully recovered (56.8% vs 40.1%, p<0.001), fever (20.3% vs 2.0%, p<0.001) and experiencing at least one persistent or new symptom (69.7% vs 42.4 %, p<0.001) compared with in LMICs. This was also reflected in the QoL and psychosocial assessment; a significantly higher proportion of participants in HICs reported depression/anxiety (11.0% vs 6.3%, p<0.001), pain/discomfort (11.9% vs 7.0%, p<0.001) and having problems doing usual activities (10.6% vs 3.8%, p<0.001) compared with those in LMICs.

**Figure 2:**
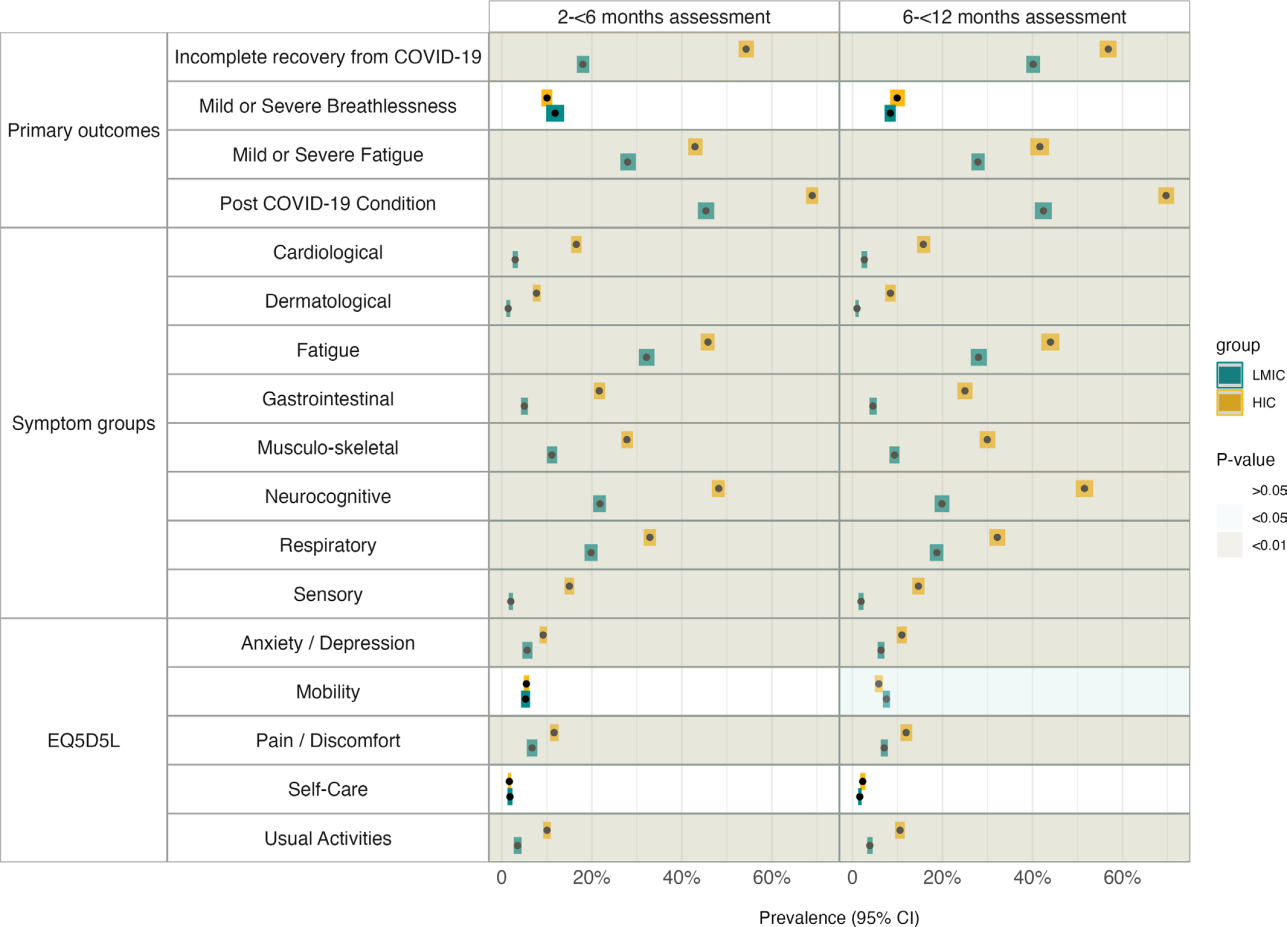
Post-COVID-19 sequelae post-hospital discharge in HICs compared with LMICs. The figures present the differences in post-COVID-19 manifestations among participants in lower-to-middle-income settings (blue dots) compared with those in high-income settings (orange dots). The grey shading illustrates the level of significance, dark grey=p< 0.01, medium grey=p<0.05, white=non-significant differences. EQ5D5L, EuroQol 5-Dimension 5-level; HICs, high-income countries; LMIC, lower-income and middle-income countries.

When grouping symptoms into physiological symptom clusters, all clusters were significantly more common in the study population in HICs versus LMICs at both time points. Neurocognitive symptoms were the most commonly reported (48.2% vs 21.8%, p<0.001) at 2 to <6 months; (51.5% vs 19.9%, p<0.001) at 6 to <12 months in HIC vs LMIC), followed by respiratory symptoms (32.9% vs 19.8%, p<0.001 at 2 to <6 months; 32.2% vs 18.7%, p<0.001 at 6 to <12 months in HICs vs LMICs) (figure 2, online supplemental file 4:table S2)

### Breathlessness

**Figure 3:**
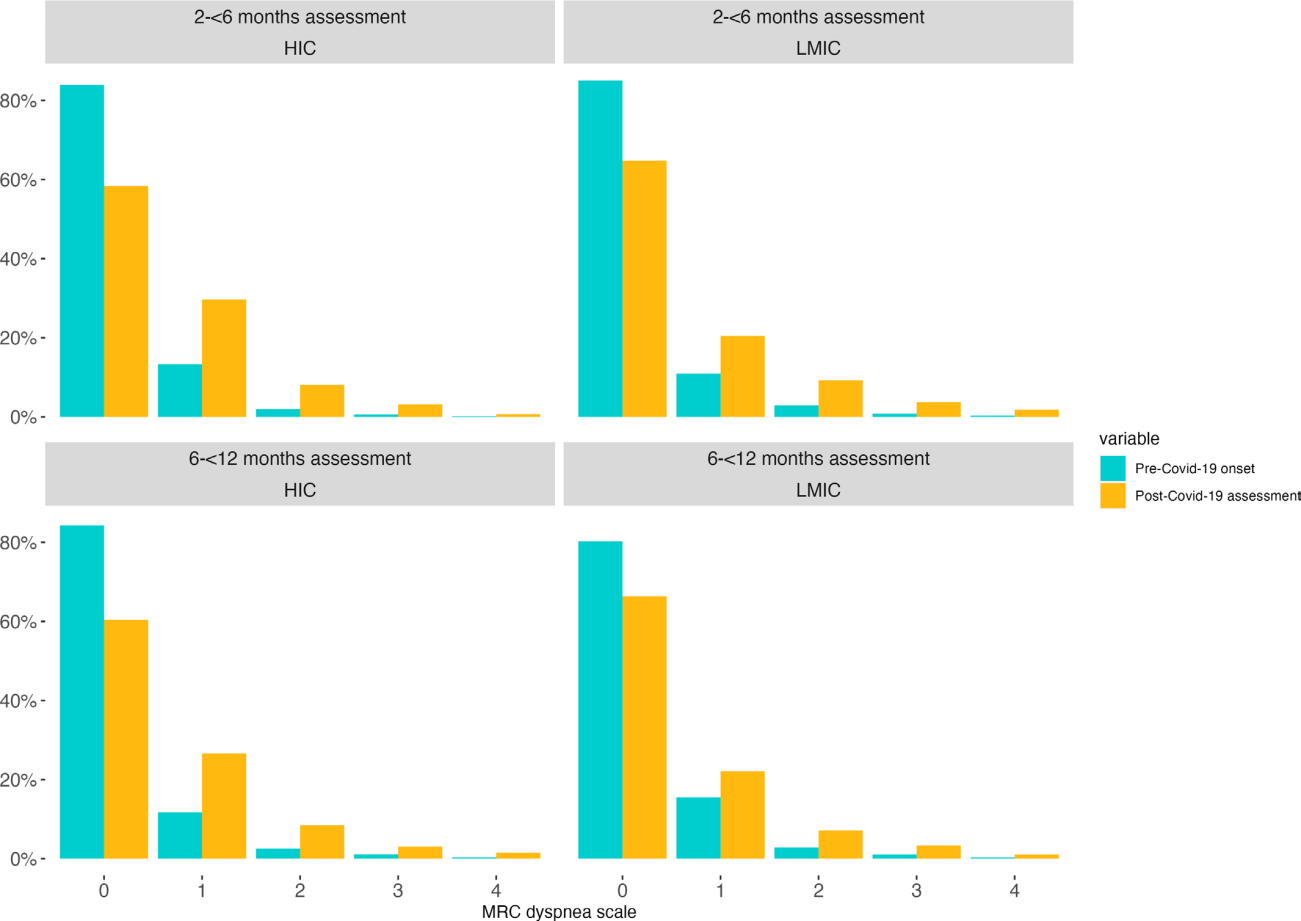
Breathlessness before and after SARS-CoV-2 infection by setting. The graph shows the level of breathlessness before and after developing COVID-19 measured using the Medical Research Council (MRC) dyspnoea scale. 0=no breathlessness; 1=mild breathlessness, 2=breathlessness, 3=moderate breathlessness; 4=severe breathlessness. HIC, high-income country; LMIC, lower-income and middle-income country.

There was a significant increase in the proportion of people experiencing breathlessness at the follow-up assessment compared with prior to their COVID-19 illness. Although the frequency was lower at the 6 to 12 months assessment compared with the earlier assessment, a proportion had still not recovered to pre-COVID-19 levels (figure 3). The most considerable deterioration was observed in breathlessness dynamics: the proportion of those whose breathlessness worsened after COVID-19 is larger than those whose breathlessness remained the same or improved.

### Risk factors for developing postacute COVID-19 physical sequelae

**Figure 4:**
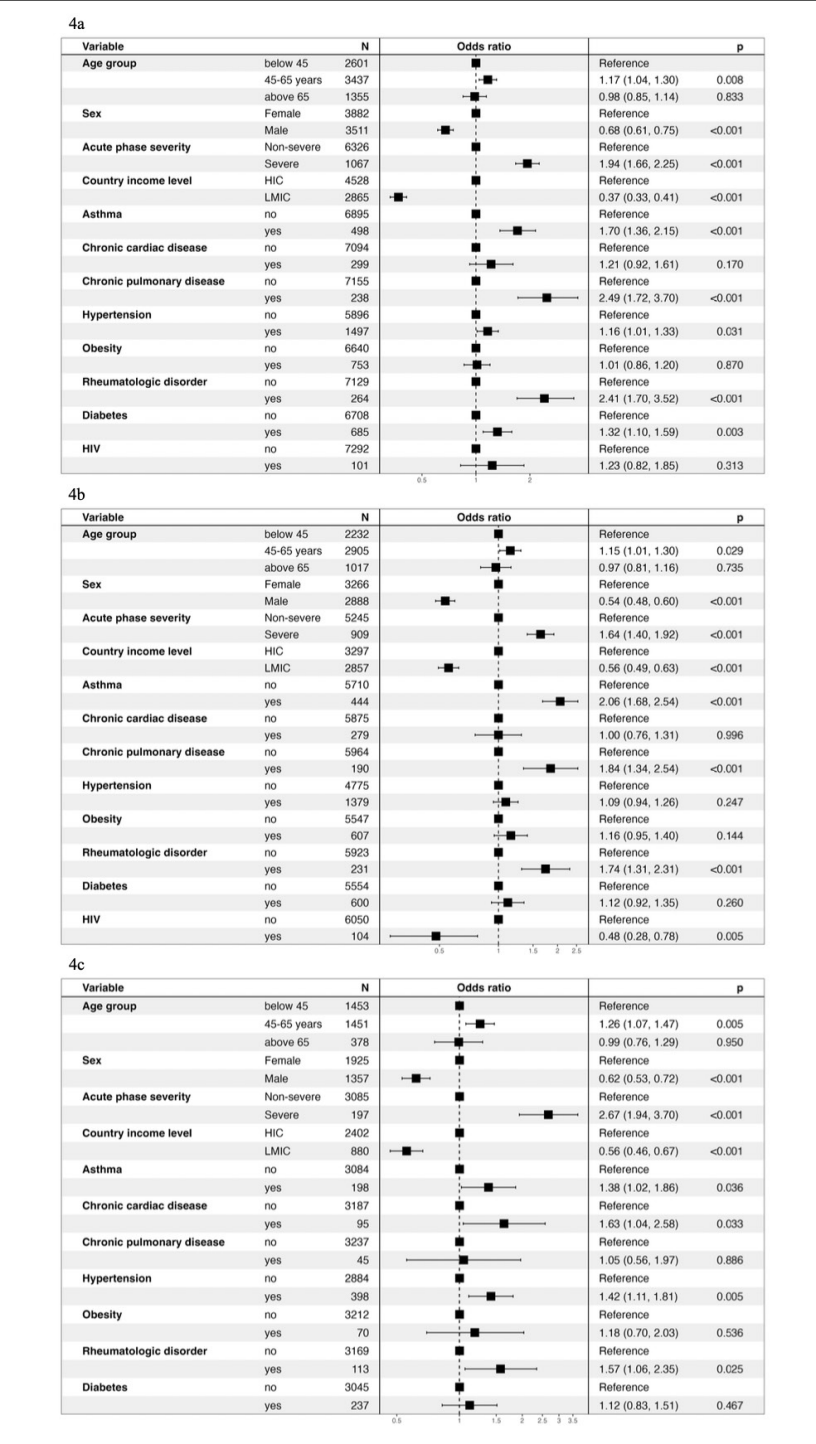
Risk factors for long -term COVID-19 sequelae The graphs present risk factors for experiencing Long Covid (**4a**), fatigue (**4b**) and reduced functioning and quality of life (QoL) (**4c**), respectively, at 2 -to- < 6 months post-COVID-19 hospital discharge. HIC: high-income country; LMIC: lower-income and middle-income country.

Patients with severe acute COVID-19 during hospitalisation had a higher risk of developing Long Covid, moderate-to-severe fatigue, decreased functioning and QoL at 2 to <6 months follow-up than patients with moderate acute COVID-19 (figure 4a–c). Males had a lower risk of developing Long Covid, moderate-to-severe fatigue, with reduced functioning and QoL than females. Participants from LMICs had a lower risk of developing Long Covid, moderate-to-severe fatigue, with decreased functioning and QoL compared with participants in HICs. Being 45–65 years old was associated with a significantly increased risk of experiencing reduced functioning and QoL compared with younger adults. Pre-existing asthma, chronic pulmonary and rheumatological disease were identified as significant risk factors for developing Long Covid and fatigue, hypertension and chronic cardiac disease were additional risk factors for experiencing reduced functioning and QoL.

## Discussion

Our data show that Long Covid affects populations globally, manifesting with similar symptomatology and impact on functioning and QoL. Although the frequency of Long Covid was higher in countries in high-income settings, the impact on individuals may be higher in lower-resourced settings where the healthcare and socioeconomic support infrastructure may be more limited. Our data highlight an urgent need for investment into research, care and support, to identify underlying pathophysiology inform treatment and trials and identify effective and accessible treatment strategies to improve long-term COVID-19 outcomes. The higher rates of persistent symptoms, fatigue and reduced functioning observed in participants from HICs compared with LMICs may be explained by multiple factors. Differences in awareness of the condition, limited healthcare access and ability to seek medical care in lower-resourced settings may lead to underdiagnosis and under-reporting of long-term COVID-19 sequelae. Cultural differences in how symptoms are perceived and reported may also contribute to reporting biases. The differences may also be due to varying underlying pandemic trajectories and viral factors such as the dominating circulating strain at different time points and settings. Later circulating SARS-CoV-2 strains, such as Omicron, were associated with a lower risk of COVID-19 sequelae than earlier pandemic strains.^30^ The first sites to establish Long Covid cohorts in this study were in HICs, which may bias the inclusion of people with early SARS-CoV-2 strains in these settings.^31^ In addition, the higher rates of risk factors identified, such as hypertension and asthma in HICs, may also play a role. Due to the complexity of recording ethnicity in large international studies due to the rich, diverse ethnic groups in a globalised world, it was not possible to draw any conclusions on the association between ethnicity and the risk of developing Long Covid. Further research is needed to explore the differences in Long Covid burden observed and explore the impact of host and pathogen factors.

The most commonly reported symptoms were fatigue/malaise, headache, shortness of breath and muscle/joint pain. Neurocognitive symptoms were the most common when grouping symptoms into clusters, followed by respiratory symptoms. These findings are in agreement with other studies.^16 17^ Prolonged recovery has featured in other postviral and postintensive care syndrome studies, describing some commonality in symptoms such as breathlessness, fatigue, muscle weakness and impact on QoL. However, these studies identified a lower frequency of postviral complications than post-SARS-CoV-2 studies. This may partly be explained by the challenges in comparing patients with different disease severity. When disease severity is similar to when comparing classic acute respiratory distress syndrome (ARDS) versus COVID-19-associated ARDS (CARDS), a study identified that persisting disability may be more common in survivors of classic ARDS compared with CARDS.^32^ However, cognitive and psychological health status may be relatively less frequently impaired than physical functioning.^33^

Interestingly, in contrast to the other outcomes with diverse frequencies between study populations and over time, there were no significant differences in the proportion of participants with moderate-to-severe breathlessness between LMICs and HICs, nor assessment time points. Approximately 1 in 10 participants was affected by moderate-to-severe breathlessness, which aligns with global estimates of Long Covid. Breathlessness was assessed using a validated, well-recognised and tested tool, the MRC dyspnoea scale, recognised as the key instrument for measuring core outcome sets in Long Covid studies.^34^

Our data illustrate the importance of implementing standardised and validated tools to identify reliable data and facilitate comparative analysis. The reason for the differences in presentation may relate to wider factors as described earlier, but it also suggests that there may be different underlying aetiology, such as specific organ damage, persistent, infection-triggered autoimmunity and dysautonomia behind different clusters of symptoms. Breathlessness, may be related to direct lung or cardiovascular organ damage, cellular gas exchange and/or diaphragm muscle impairment. Radiological Long Covid abnormalities have been identified in various organs, including the olfactory bulb, brain, heart and lungs^35^ Micro clots and hypercoagulation biomarkers indicating endothelial activation and abnormal clotting have been implicated as causative agents for some Long Covid symptoms.^35^ The high proportion of people with new or persistent fever identified in our study might be explained by a persistent infection. Both persistent SARS-CoV-2 infection and reactivation of Epstein-Barr virus have been proposed as possible underlying causative agents of long COVID.^35 36^ Further research is needed to identify the underlying pathophysiological mechanisms to guide treatment trials and provide more precise case definitions to guide diagnostic and care referral pathways and inclusion in trials.

The proportion of people reporting newly developed or persistent symptoms not experienced before onset of their COVID-19 illness was higher than those reporting not feeling fully recovered. This indicates that existing Long Covid definitions are too broad, lacking the precision needed to guide clinical and public health management. This poses a risk of Long Covid being misdiagnosed, with associated risks and impact on patients and health systems from both overdiagnosis and underdiagnosis.

This study has limitations: it was not possible to follow up with all possible participants discharged from the study hospitals due to a combination of factors, including pandemic resource constraints, contact details not being available, patients not responding to the invitation or not consenting to participate. Although efforts were made to facilitate inclusion, such as using multiple contact methods (mail, telephone and online) in different languages, it may be that non-responders were too unwell to respond, did not receive the invite, had died or moved away, had recovered or were not interested in participating. Therefore, the results may not fully represent the population of people hospitalised with COVID-19 in the participating hospitals and countries. This potential completion bias may mean that the data is underestimating or overestimating the prevalence. Further, patients were asked to retrospectively rate their health status before their COVID-19 onset, which may be impacted by recall bias. The different data collection methods (clinician-led assessment in the clinic and by phone or online) designed for broad uptake during the pandemic constraints and cultural interpretation of the questions may add heterogeneity. Due to resource and pandemic constraints, we could not include a control group of participants hospitalised with a non-COVID-19 illness. As the pandemic progressed through waves of new and more transmissible strains, identifying and ascertaining meaningful controls became increasingly difficult. Therefore, it was not possible to fully assess pandemic-related factors impacting QoL. However, similar symptoms presenting in different countries during the study period indicate that pandemic restrictions are not the likely aetiology. Finally, due to the different ethnicity definitions in countries, and regulations in some countries preventing documentation of ethnicity, a sensitive analysis based on different ethnicities was not feasible.

Despite these limitations, this is, to our knowledge, the largest international study on Long Covid using a standardised data collection form and predominantly clinician-led assessments, enabling characterisation and comparison of Long Covid and its impact on functioning in participants following COVID-19 hospitalisation in different resourced settings globally.

Reflecting on this study, similar research in future could be improved by (1) developing a pathogen-blind protocol including appropriate control groups, hibernating studies and making logistical provision for swift deployment of a study for future epidemics and pandemics; (2) collecting information about postinfection sequelae in both hospitalised and community cohorts in a single study to facilitate comparison between these groups; (3) linking postinfection studies to studies of the acute illness to facilitate improved data linkage and reduce loss to follow-up and (4) consider strategies to include poorly represented groups such as low-income countries, rural communities, children, the elderly and any minority ethnic groups within countries.

The data show that a significant proportion of adults hospitalised with COVID-19 in HICs and LMICs are impacted by long-term sequelae 1-year postdischarge, presenting with similar symptoms, illustrating that Long Covid is a universal health issue. The WHO Post-Covid Condition definition is broad and does not distinguish between different underlying pathophysiology. Our data show the urgent need to define Long Covid clusters, to guide clinical referral pathways and inclusion in trials.

The wide range of symptoms identified indicates that patients may present to multiple specialities within the healthcare system. Highlighting the need to establish clear referral pathway guidelines accessible in front-line primary and secondary care clinics, supported by access to appropriate diagnostics and treatments. Despite the global research effort during the pandemic, most funding was channelled into acute trials. Many research priorities identified at an international stakeholder forum organised by the WHO and GloPID-R in December 2020 remain unanswered, largely due to a lack of investment into and coordination of research during the pandemic.^37^ In the future, we need to integrate standardised follow-up protocols into clinical studies and adaptive trial platforms to strengthen our capacity for timely data generation to inform the clinical and public health response.

## Conclusions

Our study shows that Long Covid affects populations globally, with a significant proportion of people experiencing persistent symptoms impacting on functioning and QoL a year after COVID-19 onset. The data emphasise the potential long-term impact on population health globally, which may disproportionately impact economically disadvantaged populations.

In HICs, the prevalence of Long Covid is higher. This may reflect better awareness, along with a broader range of reported symptoms, including significant neurocognitive and psychosocial issues. This underscores the importance of accessible and comprehensive healthcare systems, as well as robust mental health and social support. In LMICs, the lower reported prevalence may suggest under-reporting due to limited healthcare access and resources, however, the proactive follow-up that this study required may have ameliorated these issues.

Long Covid may have a disproportionately severe impact on daily functioning in LMICs, compounded by inadequate healthcare infrastructure and higher rates of pre-existing conditions.

There is an urgent need for significant investment in healthcare capacity building in both HICs and LMICs to improve diagnosis, management and support for Long Covid. As COVID-19 becomes endemic, continuing to infect and reinfect people worldwide, further investments in identifying effective prevention and treatment of acute and long-term complications accessible to all populations are essential to improve outcomes.

## Supplementary material

10.1136/bmjgh-2024-015245 Supplementary file 1

## Data Availability

Data are available on reasonable request.
