# Supplementary material for: Long Covid: a global health issue – a prospective, cohort study set in four continents
Source: BMJ Glob Health. 2024 Oct 21;9(10):e015245. doi: 10.1136/bmjgh-2024-015245 (PMC11552006; doi:10.1136/bmjgh-2024-015245)
Supplement: Supplementary file 1 [file bmjgh-9-10-s001.pdf]

Supplementary file 1: Box S1: Physiological and systemic clusters

- **Cardiological:** palpitations
- **Dermatological:** skin rash, Covid-toes
- **Fatigue:** fatigue
- **Fever:** measured or self-reported
- **Gastrointestinal:** abdominal pain, diarrhoea, constipation, vomiting / nausea;
- **Musculoskeletal:** muscle aches / joint pain, ankle swelling;
- **Neuro-cognitive:** headache, problems with sleeping, altered consciousness / confusion, dizziness, tinnitus, problems with balance, paraesthesia, tremor, problems with speaking or communicating, fainting / blackouts, loss of sensation, seizures;
- **Respiratory:** cough, cough productive, cough non-productive, shortness of breath, pain on breathing, chest pain;
- **Sensory:** lost or altered sense of smell, lost or altered sense of taste

Supplemental file 2: Figure S1. STROBE Flowchart

*n= 2285 participants participated in both follow-up assessments

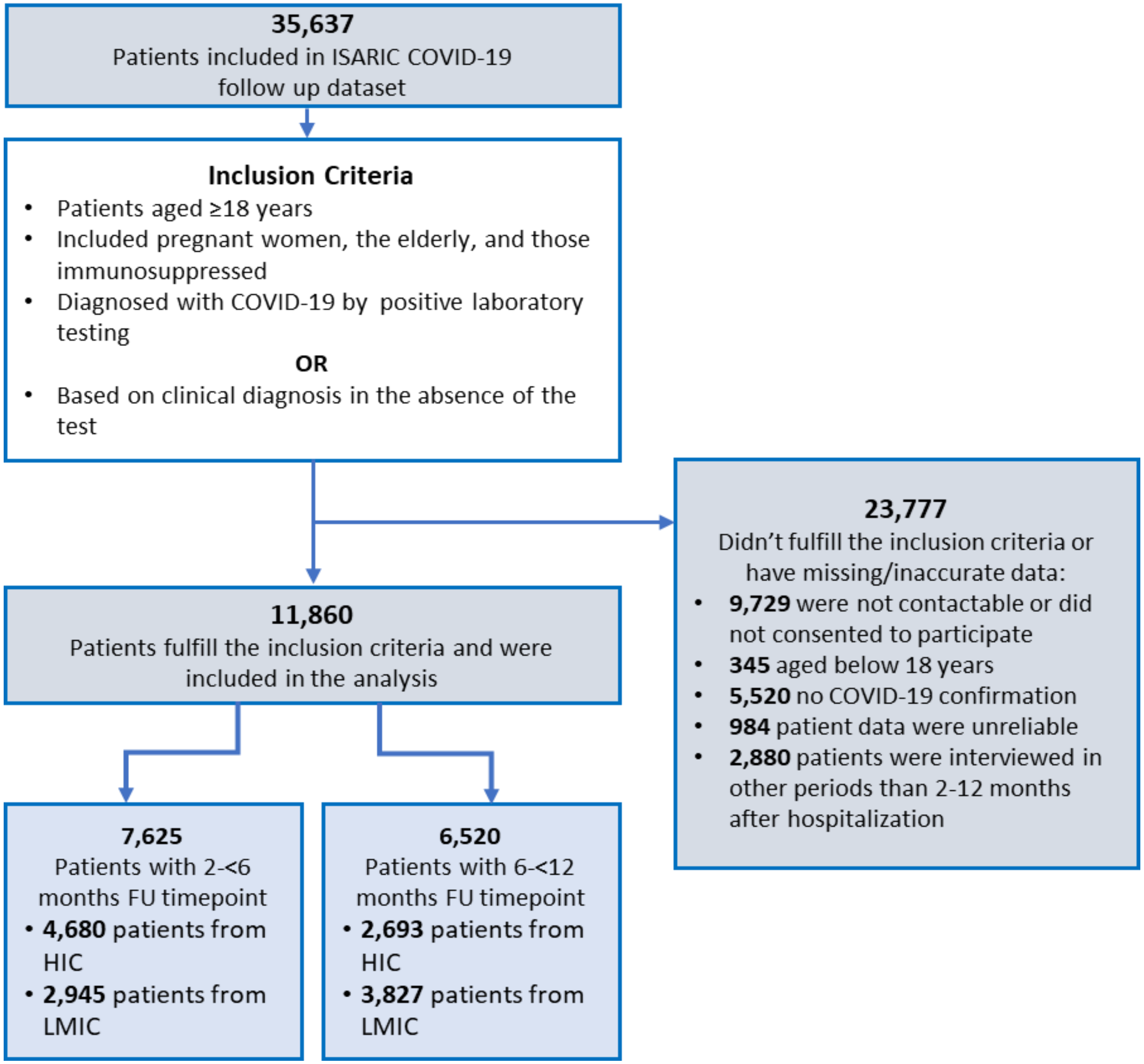

Supplementary file 3: Table S1: Cohort description by country

Characteristics	Total N = 11860	BR N = 444	CO N=173	FR N= 384	GM N=29	GI N=390	IN N=918	IL N=930	IT N=257	MY N=26	NO N= 2487	PT N= 23	RU N=1343	ZA N=2198	ES N=694	SD N = 1	UA N= 30	GB N= 1533
**Age**	52.0 (41.0, 62.0) / 11860.0	42 (32,53) / 444	57 (46,65) / 173	59 (48,69) / 384	57 (43,66) / 29	48 (36,57) / 390	50 (38,60) / 918	44 (35,57) / 930	50 (44,65) / 257	54 (39,61) / 26	46 (35,55) / 2,487	61 (54,70) / 23	58 (47,67) / 1,343	52 (41,61) / 2,198	60 (49,71) / 694	30 (30,30) / 1	66 (56,72) / 30	59 (51,68) / 1,533
**Gender**																		
**Female**	6147 (51.8%)	299 (67.6%)	64 (37%)	134 (35%)	7 (24.1%)	238 (61.2%)	295 (32.1%)	621 (68.1%)	118 (46.6%)	11 (42.3%)	1598 (65.1%)	4 (17.4%)	676 (50.3%)	1130 (51.4%)	325 (46.8%)	1 (100%)	19 (63.3%)	607 (39.6%)
**Male**	5655 (47.7%)	143 (32.4%)	109 (63%)	249 (65%)	22 (75.9%)	151 (38.8%)	623 (67.9%)	291 (31.9%)	135 (53.4%)	15 (57.7%)	858 (34.9%)	19 (82.6%)	667 (49.7%)	1068 (48.6%)	369 (53.2%)	0 (0%)	11 (36.7%)	925 (60.4%)
**Unknown**	58 (0.5%)	2 (0.5%)	0 (0%)	1 (0.3%)	0 (0%)	1 (0.3%)	0 (0%)	18 (2%)	4 (1.6%)	0 (0%)	31 (1.3%)	0 (0%)	0 (0%)	0 (0%)	0 (0%)	0 (0%)	0 (0%)	1 (0.1%)
**Ethnicity**																		
**Asian**	1297 (10.9%)	1 (0.2%)	NA	NA	NA	NA	NA	0 (0%)	NA	NA	61 (2.5%)	0 (0%)	0 (0%)	156 (7.1%)	9 (1.3%)	0 (0%)	0 (0%)	156 (10.2%)
**Black**	1499 (12.6%)	71 (16%)	NA	NA	NA	NA	NA	0 (0%)	NA	NA	35 (1.4%)	0 (0%)	0 (0%)	1249 (56.8%)	4 (0.6%)	1 (100%)	0 (0%)	97 (6.3%)
**Latin American**	475 (4%)	74 (16.7%)	NA	NA	NA	NA	NA	0 (0%)	NA	NA	19 (0.8%)	0 (0%)	0 (0%)	0 (0%)	223 (32.1%)	0 (0%)	0 (0%)	2 (0.1%)
**Other ethnicities**	265 (2.2%)	0 (0%)	NA	NA	NA	NA	NA	110 (11.8%)	NA	NA	28 (1.1%)	0 (0%)	0 (0%)	3 (0.1%)	0 (0%)	0 (0%)	0 (0%)	91 (5.9%)
**White**	5751 (48.5%)	127 (28.6%)	NA	NA	NA	NA	NA	356 (38.3%)	NA	NA	2,289 (92%)	22 (95.7%)	0 (0%)	780 (35.5%)	450 (64.8%)	0 (0%)	30 (100%)	1,157 (75.5%)
**Unknown**	2573 (21.7%)	171 (38.5%)	17 (9.8%)	298 (77.6%)	0 (0%)	4 (1%)	39 (4.2%)	464 (49.9%)	133 (51.8%)	0 (0%)	55 (2.2%)	1 (4.3%)	1343 (100%)	10 (0.5%)	8 (1.2%)	0 (0%)	0 (0%)	30 (2%)
**Severity during acute phase**																		
Moderate	10353 (87.3%)	439 (98.9%)	135 (78%)	268 (69.8%)	29 (100%)	383 (98.2%)	879 (95.8%)	886 (95.3%)	240 (93.4%)	25 (96.2%)	2403 (96.6%)	0 (0%)	1,314 (97.8%)	1604 (73%)	635 (91.5%)	1 (100%)	30 (100%)	1082 (70.6%)
Severe patients	1507 (12.7%)	5 (1.1%)	38 (22%)	116 (30.2%)	0 (0%)	7 (1.8%)	39 (4.2%)	44 (4.7%)	17 (6.6%)	1 (3.8%)	84 (3.4%)	23 (100%)	29 (2.2%)	594 (27%)	59 (8.5%)	0 (0%)	0 (0%)	451 (29.4%)
Days from hospital admission to follow up	164.5 (126.0, 239.0) / 6815	NA (NA,NA) / 0	NA	118 (97,145) / 61	NA (NA,NA) / 0	198 (163,260) / 339	NA	NA (NA,NA) / 0	169 (131,204) / 255	142 (132,148) / 5	149 (112,187) / 2,448	68 (65,84) / 18	NA (NA,NA) / 0	NA	NA (NA,NA) / 0	NA (NA,NA) / 0	62 (62,63) / 30	220 (185,274) / 270
**Pre-existing risk comorbidities and risk factors**																		
**Asthma**	798 / 11,860 (6.7%)	13 / 444 (2.9%)	1 / 173 (0.6%)	39 / 384 (10%)	0 / 29 (0%)	31 / 390 (7.9%)	23 / 918 (2.5%)	30 / 930 (3.2%)	9 / 257 (3.5%)	1 / 26 (3.8%)	192 / 2,487 (7.7%)	2 / 23 (8.7%)	72 / 1,343 (5.4%)	84 / 2,198 (3.8%)	6 / 694 (0.9%)	0 / 1 (0%)	1 / 30 (3.3%)	294 / 1,533 (19%)
**Chronic cardiac disease not hypertension**	707 / 11,860 (6.0%)	5 / 444 (1.1%)	1 / 173 (0.6%)	59 / 384 (15%)	0 / 29 (0%)	9 / 390 (2.3%)	16 / 918 (1.7%)	8 / 930 (0.9%)	28 / 257 (11%)	0 / 26 (0%)	65 / 2,487 (2.6%)	5 / 23 (22%)	268 / 1,343 (20%)	66 / 2,198 (3.0%)	1 / 694 (0.1%)	0 / 1 (0%)	26 / 30 (87%)	150 / 1,533 (9.8%)
**Chronic pulmonary**	463 / 11,860	3 / 444 (0.7%)	2 / 173 (1.2%)	20 / 384	1 / 29 (3.4%)	3 / 390 (0.8%)	2 / 918 (0.2%)	12 / 930	8 / 257 (3.1%)	1 / 26 (3.8%)	37 / 2,487	6 / 23 (26%)	109 / 1,343	21 / 2,198	4 / 694 (0.6%)	0 / 1 (0%)	2 / 30 (6.7%)	232 / 1,533

**disease not asthma**	(3.9%)			(5.2%)				(1.3%)			(1.5%)		(8.1%)	(1.0%)				(15%)
**HIV**	377 / 11,860 (3.2%)	0 / 444 (0%)	0 / 173 (0%)	1 / 384 (0.3%)	1 / 29 (3.4%)	0 / 390 (0%)	0 / 918 (0%)	0 / 930 (0%)	0 / 257 (0%)	0 / 26 (0%)	5 / 2,487 (0.2%)	0 / 23 (0%)	264 / 1,343 (20%)	103 / 2,198 (4.7%)	0 / 694 (0%)	0 / 1 (0%)	0 / 30 (0%)	3 / 1,533 (0.2%)
**Hypertension**	2,650 / 11,860 (22%)	97 / 444 (22%)	10 / 173 (5.8%)	134 / 384 (35%)	11 / 29 (38%)	21 / 390 (5.4%)	200 / 918 (22%)	68 / 930 (7.3%)	58 / 257 (23%)	8 / 26 (31%)	182 / 2,487 (7.3%)	10 / 23 (43%)	640 / 1,343 (48%)	709 / 2,198 (32%)	12 / 694 (1.7%)	0 / 1 (0%)	21 / 30 (70%)	469 / 1,533 (31%)
**Obesity**	1,190 / 11,860 (10%)	0 / 444 (0%)	1 / 173 (0.6%)	45 / 384 (12%)	2 / 29 (6.9%)	0 / 390 (0%)	0 / 918 (0%)	0 / 930 (0%)	23 / 257 (8.9%)	2 / 26 (7.7%)	62 / 2,487 (2.5%)	10 / 23 (43%)	263 / 1,343 (20%)	600 / 2,198 (27%)	130 / 694 (19%)	0 / 1 (0%)	4 / 30 (13%)	48 / 1,533 (3.1%)
**Rheumatologic al disorder**	429 / 11,860 (3.6%)	6 / 444 (1.4%)	0 / 173 (0%)	9 / 384 (2.3%)	0 / 29 (0%)	10 / 390 (2.6%)	4 / 918 (0.4%)	0 / 930 (0%)	11 / 257 (4.3%)	1 / 26 (3.8%)	126 / 2,487 (5.1%)	4 / 23 (17%)	42 / 1,343 (3.1%)	0 / 2,198 (0%)	1 / 694 (0.1%)	0 / 1 (0%)	3 / 30 (10%)	212 / 1,533 (14%)
**Tuberculosis**	34 / 11,860 (0.3%)	1 / 444 (0.2%)	0 / 173 (0%)	4 / 384 (1.0%)	1 / 29 (3.4%)	0 / 390 (0%)	2 / 918 (0.2%)	0 / 930 (0%)	0 / 257 (0%)	0 / 26 (0%)	1 / 2,487 (<0.1%)	0 / 23 (0%)	1 / 1,343 (<0.1%)	23 / 2,198 (1.0%)	1 / 694 (0.1%)	0 / 1 (0%)	0 / 30 (0%)	0 / 1,533 (0%)

Abbreviations: BR (Brazil), CO (Colombia), FR (France), GM (Gambia), GI (Gibraltar), IN (India), IL (Israel), IT (Italy), MY (Malaysia), NO (Norway), PT (Portugal), RU (Russian Federation), ZA (South Africa), ES (Spain), SD (Sudan), UA (Ukraine), GB (United Kingdom)

Supplemental file 4: Figure S2. Prevalence of symptoms by setting and acute COVID-19 severity

Yellow bars: Prevalence in HIC study populations
Blue bars: Prevalence in LMIC study populations

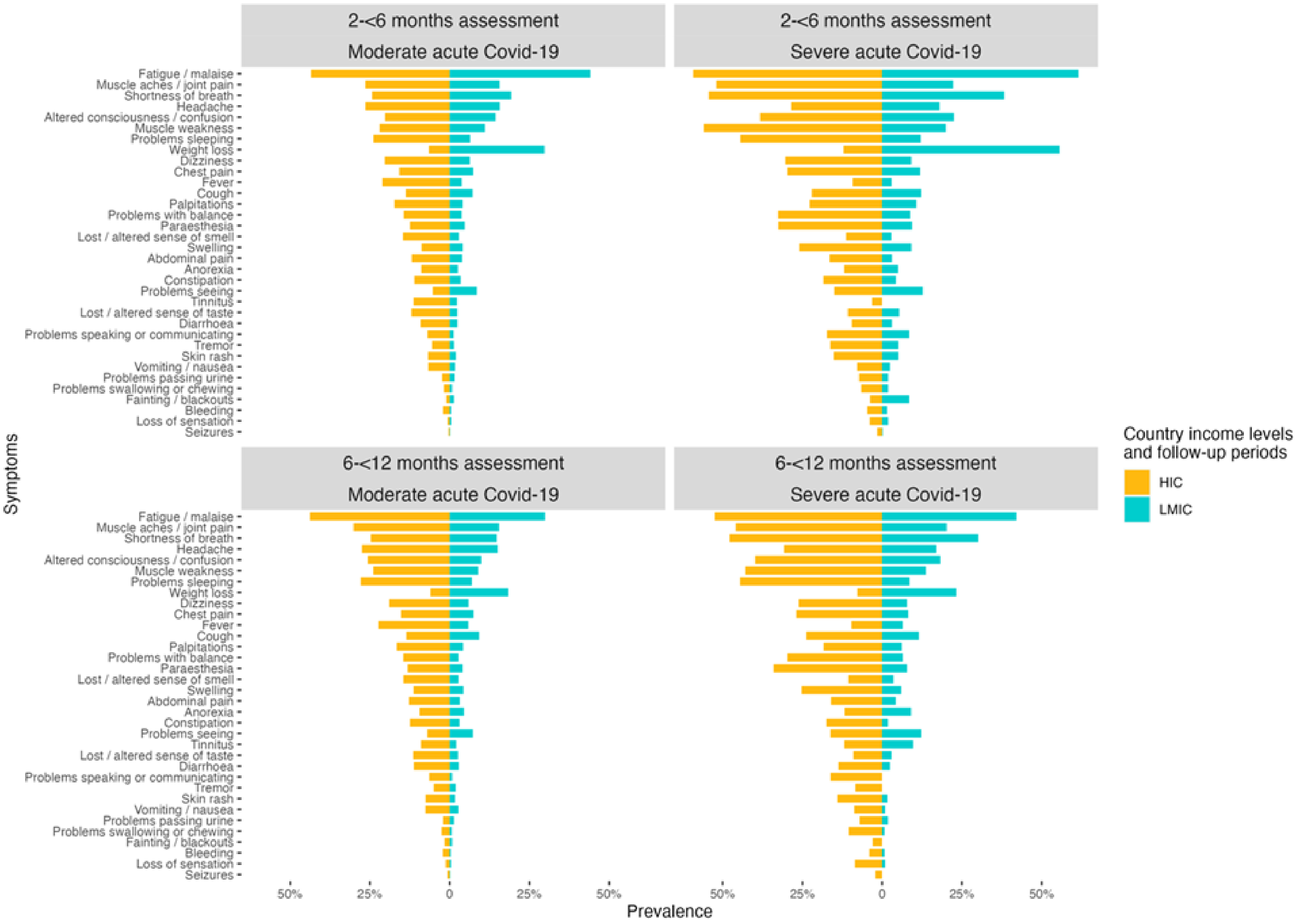

Supplemental file 5: Table S2. Prevalence of outcomes by assessment timepoint and country income levels

The respondents (n=2285) followed up at both intervals are included under each assessment time-point.

**Characteristics**	**2-<6 months assessment**			**6-<12 months assessment**		
	**HIC**	**LMIC**	**p-value**	**HIC**	**LMIC**	**p-value**
	N = 4680	N = 2945		N=2693	N=3827	
**Primary outcomes**						
**Incomplete recovery**	**54.3% (52.6% - 55.8%) / 3467**	**18.0% (16.6% - 19.4%) / 2935**	**<0.001**	**56.8% (54.9% - 58.7%) / 2366**	**40.1% (38.6% - 41.8%) / 3786**	**<0.001**
Breathlessness Moderate-severe	10.0% (8.8% - 11.2%) / 2401	11.8% (9.9% - 13.8%) / 997	0.13	9.9% (8.3% - 11.6%) / 1341	8.4% (7.2% - 9.5%) / 1964	0.13
Fatigue Moderate-severe	42.9% (41.3% - 44.5%) / 3321	27.9% (26.3% - 29.6%) / 2857	<0.001	41.6% (39.7% - 43.6%) / 2212	27.9% (26.5% - 29.3%) / 3357	<0.001
Long Covid	69.0% (67.6% - 70.3%) / 4557	45.3% (43.5% - 47.1%) / 2865	<0.001	69.7% (68.0% - 71.4%) / 2566	42.4% (40.5% - 44.5%) / 2515	<0.001
**Physiological symptom groups**						
Cardiological	16.5% (15.4% - 17.7%) / 4068	3.0% (2.4% - 3.6%) / 2803	<0.001	15.7% (14.2% - 17.4%) / 2420	2.6% (2.0% - 3.3%) / 2471	<0.001
Dermatological	7.7% (6.8% - 8.6%) / 3514	1.4% (1.0% - 1.9%) / 2843	<0.001	8.4% (7.4% - 9.6%) / 2301	1.0% (0.6% - 1.4%) / 2499	<0.001
Fatigue	45.8% (44.2% - 47.3%) / 3895	32.1% (30.3% - 34.0%) / 2813	<0.001	44.0% (41.9% - 46.0%) / 2439	28.0% (26.2% - 29.8%) / 2469	<0.001
Fever	19.6% (18.1% - 21.1%) / 2974	2.1% (1.6% - 2.6%) / 2835	<0.001	20.3% (18.6% - 22.3%) / 1687	2.0% (1.5% - 2.6%) / 2499	<0.001
Gastrointestinal	21.6% (20.4% - 22.9%) / 4117	5.0% (4.2% - 5.9%) / 2800	<0.001	25.0% (23.2% - 26.7%) / 2430	4.5% (3.7% - 5.4%) / 2470	<0.001
Musculoskeletal	27.8% (26.4% - 29.1%) / 4236	11.1% (10.0% - 12.3%) / 2791	<0.001	29.9% (28.1% - 31.7%) / 2521	9.3% (8.2% - 10.5%) / 2451	<0.001
Neurocognitive	48.2% (46.7% - 49.6%) / 4526	21.8% (20.2% - 23.3%) / 2830	<0.001	51.5% (49.6% - 53.4%) / 2551	19.9% (18.3% - 21.5%) / 2481	<0.001
Respiratory	32.9% (31.6% - 34.3%) / 4491	19.8% (18.4% - 21.4%) / 2831	<0.001	32.2% (30.4% - 34.1%) / 2548	18.7% (17.3% - 20.3%) / 2481	<0.001
Sensory	15.0% (13.9% - 16.2%) / 3659	2.0% (1.5% - 2.5%) / 2824	<0.001	14.6% (13.2% - 16.1%) / 2418	1.9% (1.4% - 2.4%) / 2476	<0.001
Impact on functioning, psychosocial symptoms and quality of life						
Anxiety / Depression	9.2% (8.4% - 10.0%) / 4928	5.6% (4.6% - 6.7%) / 1780	<0.001	11.0% (9.9% - 12.1%) / 2734	6.3% (5.5% - 7.1%) / 3942	<0.001
Mobility	5.5% (4.8% - 6.1%) / 4954	5.3% (4.3% - 6.3%) / 1784	0.47	5.8% (5.0% - 6.7%) / 2742	7.5% (6.7% - 8.4%) / 3958	0.01**
Pain / Discomfort	11.6% (10.7% - 12.5%) / 4928	6.7% (5.5% - 7.9%) / 1788	<0.001	11.9% (10.7% - 13.2%) / 2720	7.0% (6.3% - 7.9%) / 3946	<0.001
Self-care	1.7% (1.3% - 2.0%) / 4938	1.8% (1.2% - 2.5%) / 1780	0.49	2.3% (1.7% - 2.8%) / 2736	1.6% (1.2% - 2.0%) / 3948	0.06
Usual activities	10.0% (9.2% - 10.9%) / 4940	3.5% (2.7% - 4.4%) / 1780	<0.001	10.6% (9.4% - 11.7%) / 2744	3.8% (3.3% - 4.5%) / 3952	<0.001

Supplementary file 6: Table S3 – Sensitivity analysis for logistic regressions

Variables	Fatigue	Recovery	EQ-5D summary index change
(Intercept)	3.36 (0.11)***	4.61 (0.14)***	-0.06 (0.01)***
Age group (above 55 years)	0.1 (0.09)	-0.26 (0.07)***	0.01 (0.01).
Sex (male)	-0.67 (0.08)***	0.05 (0.06)	0.02 (0.01)**
Severity (severe)	0.28 (0.09)**	-0.15 (0.07)*	-0.07 (0.01)***
**Income (LMIC)**	**-1.24 (0.1)*** **	**0.75 (0.13)*** **	**0.02 (0.01)*** **
Asthma	1.04 (0.18)***	-0.4 (0.15)**	-0.04 (0.01)**
Chronic cardiac disease	0.55 (0.18)**	-0.52 (0.12)***	0.01 (0.01)
Chronic kidney disease	0.36 (0.3)	-0.38 (0.22).	-0.01 (0.02)
Chronic neurological disorder	0.8 (0.44)	-0.54 (0.24)*	-0.09 (0.02)***
Chronic pulmonary disease	0.43 (0.32)	-0.41 (0.19)*	0.01 (0.02)
HIV	-0.67 (0.24)**	0.34 (0.17)*	
Hypertension	0.11 (0.1)	-0.21 (0.07)**	-0.02 (0.01)*
Malignant neoplasm	0.54 (0.37)	-0.5 (0.24)*	0.01 (0.02)
Obesity	-0.07 (0.1)	0.03 (0.08)	-0.03 (0.01)*
Rheumatological disorder	0.15 (0.37)	-0.12 (0.29)	-0.04 (0.02)*
Tuberculosis	-0.1 (0.49)	0.55 (0.38)	
Diabetes mellitus	0.47 (0.13)***	-0.27 (0.1)*	-0.01 (0.01)

Supplemental file 7: ISARIC Clinical Characterisation Group

Laurent Abel, Amal Abrous, Younes Ait Tamlihat, Aliya Mohammed Alameen, Marta Alessi, Beatrice Alex, Kévin Alexandre, Adam Ali, Kazali Enagnon Alidjnou, Clotilde Allavena, Nathalie Allou, Claire Andrejak, Andrea Angheben, François Angoulvant, Séverine Ansart, Jean-Benoît Arlet, Elise Artaud-Macari, Jean Baptiste Assie, Johann Auchabie, Hugues Aumaitre, Adrien Auvet, Eyvind W. Axelsen, Laurène Azemar, Cecile Azoulay, Benjamin Bach, Delphine Bachelet, Claudine Badr, John Kenneth Baillie, Firouzé Bani-Sadr, Wendy S. Barclay, Marie Bartoli, Joaquín Baruch, Romain Basmaci, Jules Bauer, Alexandra Bedossa, Husna Begum, Marine Beluze, Nicolas Benech, Delphine Bergeaud, José Luis Bernal Sobrino, Giulia Bertoli, Simon Bessis, Sybille Bevilcaqua, Karine Bezulier, Krishna Bhavsar, Zeno Bisoffi, Laurent Bitker, Mathieu Blot, Laetitia Bodenes, Debby Bogaert, Anne-Hélène Boivin, Isabela Bolaños, Pierre-Adrien Bolze, Raphaël Borie, Elisabeth Botelho-Nevers, Lila Bouadma, Olivier Bouchaud, Sabelline Bouchez, Kévin Bouiller, Laurence Bouillet, Camile Bouisse, Anne-Sophie Boureau, Maude Bouscambert, Aurore Bousquet, Marielle Boyer-Besseyre, Axelle Braconnier, Sonja Hjellegjerde Brunvoll, Polina Bugaeva, Marielle Buisson, Danilo Buonsenso, Ingrid G. Bustos, Denis Butnaru, Roar Bævre-Jensen, André Cabie, Eder Caceres, Cyril Cadoz, Jose Andres Calvache, Valentine Campana, Pauline Caraux-Paz, Nicolas Carlier, Thierry Carmoi, Marie-Christine Carret, Gail Carson, Maire-Laure Casanova, Guylaine Castor-Alexandre, François-Xavier Catherine, Minerva Cervantes-Gonzalez, Anissa Chair, Catherine Chakveatze, Meera Chand, Jean-Marc Chapplain, Charlotte Charpentier, Julie Chas, Léo Chenard, Antoine Cheret, Thibault Chiarabini, Catherine Chirouze, Bernard Cholley, Marie-Charlotte Chopin, Yock Ping Chow, Barbara Wanjiru Citarella, Sara Clohisey, Gwenhaël Colin, Marie Connor, Graham S. Cooke, Hugues Cordel, Andrea Cortegiani, Grégory Corvaisier, Sandrine Couffin-Cadiergues, Roxane Courtois, Stéphanie Cousse, Juan Luis Cruz Bermúdez, Jaime Cruz Rojo, Elodie Curlier, Eric D'Ortenzio, Ana da Silva Filipe, Charlene Da Silveira, Andrew Dagens, John Arne Dahl, Jo Dalton, Etienne De Montmollin, Cristina De Rose, Thushan de Silva, Nathalie DeCastro, Alexa Debard, Marie-Pierre Debray, Romain Decours, Eve Defous, Isabelle Delacroix, Karen Delavigne, Christelle Delmas, Pierre Delobel, Elisa Demonchy, Emmanuelle Denis, Dominique Deplanque, Diane Descamps, Mathilde Desvallées, Alpha Diallo, Sylvain Diamantis, Fernanda Dias Da Silva, Kévin Didier, Jean-Luc Diehl, Vincent Dinot, Fara Diop, Alphonsine Diouf, Félix Djossou, Annemarie B. Docherty, Christl A. Donnelly, Céline Dorival, Nathalie Dournon, Thomas Drake, Amiel A. Dror, Vincent Dubee, François Dubos, Alexandre Ducancelle, Susanne Dudman, Paul Dunand, Jake Dunning, Bertrand Dussol, Xavier Duval, Anne Margarita Dyrhol-Riise, Michael Edelstein, Linn Margrete Eggesbø, Mohammed El Sanharawi, Brigitte Elharrar, Merete Ellingjord-Dale, Philippine Eloy, Isabelle Enderle, Ilka Engelmann, Vincent Enouf, Olivier Epaulard, Hélène Esperou, Marina Esposito-Farese, Manuel Etienne, Marc Fabre, Isabelle Fabre, Cameron J. Fairfield, Karine Faure, Raphaël Favory, François-Xavier Ferrand, Eglantine Ferrand Devouge, Nicolas Ferriere, Céline Ficko, William Finlayson, Thomas Flament, Tom Fletcher, Aline-Marie Florence, Victor Fomin, Erwan Fourn, Christophe Fraser, Stéphanie Fry, Valérie Gaborieau, Rostane Gaci, Jean-Charles Gagnard, Amandine Gagneux-Brunon, Linda Gail Skeie, Sérgio Gaião, Carrol Gamble, Esteban Garcia-Gallo, Noelia García Barrio, Denis Garot, Valérie Garrait, Anatoliy Gavrylov, Alexandre Gaymard, Eva Geraud, Louis Gerbaud Morlaes, Jade Ghosn, Tristan Gigante, Guillermo Giordano, Michelle Girvan, Valérie Gissot, Petr Glybochko, François Goehringer, Kyle Gomez, Marie Gominet, Yanay Gorelik, Isabelle Gorenne, Laure Goubert, Cécile Goujard, Tiphaine Goulenok, Pascal Granier, Christopher A. Green, William Greenhalf, Segolène Greffe, Fiona Griffiths, Jérémie Guedj, Martin Guego, Romain Guery, Anne Guillaumot, Laurent

Guilleminault, Thomas Guimard, Ali Hachemi, Nadir Hadri, Matthew Hall, Sophie Halpin, Rebecca Hamidfar, Bato Hammarström, Hayley Hardwick, Ewen M. Harrison, Janet Harrison, Lars Heggelund, Ross Hendry, Maxime Hentzien, Diana Hernandez, Rupert Higgins, Hikombo Hitoto, Antonia Ho, Alexandre Hoctin, Isabelle Hoffmann, Jan Cato Holter, Peter Horby, Ikram Houas, Jean-Sébastien Hulot, Samreen Ijaz, Patrick Imbert, Mariachiara Ippolito, Margaux Isnard, Mette Stausland Istre, Danielle Jaafar, Salma Jaafoura, Julien Jabot, Clare Jackson, Stéphane Jaureguiberry, Florence Jego, Synne Jenum, Cédric Joseph, Mercé Jourdain, Silje Bakken Jørgensen, Ouifiya Kafif, Florentia Kaguelidou, Sabina Kali, Karl Trygve Kalleberg, Christiana Kartsonaki, Seán Keating, Sadie Kelly, Kalynn Kennon, Younes Kerroumi, Antoine Khalil, Saye Khoo, Beathe Kiland Granerud, Anders Benjamin Kildal, Antoine Kimmoun, Eyrun Floerecke Kjetland Kjetland, Paul Klenerman, Gry Kloumann Bekken, Stephen R Knight, Arsène Kpangon, Oksana Kruglova, Galyna Kutsyna, Marie Lachatre, Marie Lacoste, Nadhem Lafhej, Marie Lagrange, Fabrice Laine, Olivier Lairez, Antonio Lalueza, Marc Lambert, Marie Langelot-Richard, Vincent Langlois, Cédric Laouénan, Samira Laribi, Delphine Lariviere, Stéphane Lasry, Odile Launay, Didier Laureillard, Yoan Lavie-Badie, Andy Law, Minh Le, James Lee, Clément Le Bihan, Cyril Le Bris, Georges Le Falher, Lucie Le Fevre, Quentin Le Hingrat, Marion Le Maréchal, Soizic Le Mestre, Gwenaël Le Moal, Vincent Le Moing, Hervé Le Nagard, Sylvie LeGac, Jennifer Lee, Gary Leeming, Laurent Lefebvre, Bénédicte Lefebvre, Benjamin Lefèvre, Jean-Daniel Lelievre, Adrien Lemaignen, Véronique Lemee, Anthony Lemeur, Marc Leone, Quentin Lepiller, François-Xavier Lescure, Olivier Lesens, Mathieu Lesouhaitier, Sophie Letrou, Bruno Levy, Yves Levy, Claire Levy-Marchal, Geoffrey Liegeon, Wei Shen Lim, Bruno Lina, Andreas Lind, Guillaume Lingas, Sylvie Lion-Daolio, Marine Livrozet, Paul Loubet, Bouchra Loufti, Guillame Louis, Jean-Christophe Lucet, Carlos Lumbreras Bermejo, Miles Lunn, Liem Luong, Dominique Luton, Moïse Machado, Gabriel Macheda, Guillermo Maestro de la Calle, Rafael Mahieu, Sophie Mahy, Mylène Maillet, Thomas Maitre, Denis Malvy, Victoria Manda, Laurent Mandelbrot, Julie Mankikian, Aldric Manuel, Samuel Markowicz, Laura Marsh, Guillaume Martin-Blondel, Martin Martinot, Olga Martynenko, Mathieu Mattei, Laurence Maulin, Thierry Mazzoni, Sarah E. McDonald, Kenneth A. McLean, Cécile Mear-Passard, France Mentré, Alexander J. Mentzer, Emmanuelle Mercier, Antoine Merckx, Mayka Mergeay-Fabre, Laura Merson, Roberta Meta, Agnès Meybeck, Alison M. Meynert, Vanina Meysonnier, Mehdi Mezidi, Isabelle Michelet, Shona C. Moore, Sarah Moore, Lina Morales Cely, Lucia Moro, Clara Mouton Perrot, Julien Moyet, Jimmy Mullaert, Daniel Munblit, Marlène Murris, Srinivas Murthy, Alamin Mustafa, Karl Erik Müller, Fredrik Müller, Ebrahim Ndure, Nadège Neant, Nikita Nekliudov, Anthony Nghi, Duc Nguyen, Mahdad Noursadeghi, Saad Nseir, Elsa Nyamankolly, Anders Benteson Nygaard, Piero L. Olliaro, Wilna Oosthuyzen, Peter Openshaw, Claudia Milena Orozco-Chamorro, Paul Otiku, Nadia Ouamara, Rachida Ouissa, Eric Oziol, Maïder Pagadoy, Justine Pages, Massimo Palmarini, Carlo Palmieri, Nathalie Pansu, Aurélie Papadopoulos, Jérémie Pasquier, Bruno Pastene, Christelle Paul, William A. Paxton, Jean-François Payen, Miguel Pedrera Jiménez, Florent Peelman, Nathan Peiffer-Smadja, Vincent Peigne, Daniel Perez, Thomas Perpoint, Vincent Pestre, Ventzislava Petrov-Sanchez, Frank Olav Pettersen, Gilles Peytavin, Walter Picard, Olivier Picone, Lionel Piroth, Chiara Piubelli, Riinu Pius, Laurent Plantier, Julien Poissy, Ryadh Pokeerbux, Georgios Pollakis, Diane Ponscarme, Sébastien Preau, Mark G. Pritchard, Víctor Quirós González, Else Quist-Paulsen, Christian Rabaud, Marie Rafiq, Blandine Rammaert, Christophe Rapp, Stanislas Rebaudet, Sarah Redl, Dag Henrik Reikvam, Anne-Sophie Resseguier, Matthieu Revest, Luis Felipe Reyes, Antonia Ricchiuto, Laurent Richier, Patrick Rispal, Karine Risso, Stephanie Roberts, David L. Robertson, Olivier Robineau, Paola Rodari, Amanda Rojek, Roberto Roncon-Albuquerque Jr, Mélanie Roriz, Manuel Rosa-Calatrava, Andrea Rossanese, Patrick Rossignol, Carine Roy, Benoît Roze, Clark D.

Russell, Aleksander Rygh Holten, Nadia Saidani, Charlotte Salmon Gandonniere, Hélène Salvator, Olivier Sanchez, Vanessa Sancho-Shimizu, Pierre-François Sandrine, Benjamine Sarton, Egle Saviciute, Arnaud Scherpereel, Marion Schneider, Janet T. Scott, Nicholas Sedillot, Malcolm G. Semple, Eric Senneville, Pablo Serrano Balazote, Catherine A. Shaw, Victoria Shaw, Nassima Si Mohammed, Jeanne Sibiude, Louise Sigfrid, Dario Sinatti, Vegard Skogen, Sue Smith, Lene Bergendal Solberg, Tom Solomon, Agnès Sommet, Albert Sotto, Edouard Soum, Elisabetta Spinuzza, Shiranee Sriskandan, Sarah Stabler, Trude Steinsvik, Birgitte Stiksrud, David Stuart, Charlotte Summers, Andrey Svistunov, Arne Søraas, Renaud Tamisier, Coralie Tardivon, Pierre Tattevin, Marie-Capucine Tellier, Olivier Terrier, Nicolas Terzi, Vincent Thibault, Simon-Djamel Thiberville, Benoît Thill, Emma C. Thomson, Mathew Thorpe, Ryan S. Thwaites, Vadim Tieroshyn, Peter S Timashev, Jean-François Timsit, Noémie Tissot, Kristian Tonby, Cécile Tromeur, Tiffany Trouillon, Jeanne Truong, Christelle Tual, Sarah Tubiana, Lance C.W. Turtle, Anders Tveita, François Téoulé, Timothy M. Uyeki, Piero Valentini, Sylvie Van Der Werf, Noémie Vanel, Charline Vauchy, Aurélie Veislinger, Fanny Vuotto, Steve Webb, Jia Wei, Murray Wham, Paul Henri Wicky, Aurélie Wiedemann, Natalie Wright, Yazdan Yazdanpanah, Cécile Yelnik, Hodane Yonis, Marion Zabbe, Maria Zambon, David Zucman.
